# Parents’ physical literacy is associated with children’s physical activity through parental support

**DOI:** 10.1186/s12889-026-27914-z

**Published:** 2026-05-28

**Authors:** Misaki Matsunaga, Masahiro Matsui, Kenta Toyama, Yuki Someya, Yujiro Kawata, Tomonori Kito, Emi Nakamura, Hiromichi Shoji, Koya Suzuki

**Affiliations:** 1 Juntendo Administration for Sports, Health and Medical Sciences, Juntendo University, Tokyo, Japan; 2 Faculty of Health and Sports Science, Juntendo University, Chiba, Japan; 3 Department of Child Psychiatry, Yokohama City University Hospital, Kanagawa, Japan; 4 Graduate School of Health and Sports Science, Juntendo University, Chiba, Japan; 5 Institute of Health and Sports Science & Medicine, Juntendo University, Chiba, Japan; 6 Department of Physical Therapy, Faculty of Health Science, Juntendo University, Tokyo, Japan; 7 Department of Pediatrics, Faculty of Medicine, Juntendo University, Tokyo, Japan

**Keywords:** Physical Literacy, Parental Support, Parent–Child Relationship, Physical Activity Promotion, Structural Equation Modeling

## Abstract

**Background:**

Physical activity provides multifaceted physical and mental health benefits for children; however, many children do not achieve the recommended levels. As children spend much of their time with their families, parental support is a key facilitator of physical activity engagement. Physical literacy is related to an individual’s physical activity, which may, in turn, affect parental support and, consequently, children’s activity levels. Nonetheless, knowledge of how parents’ physical literacy relates to parental support and children’s activities remains scarce. We aimed to examine the associations between parents’ physical literacy, parental support, and children’s physical activity. We hypothesized that parents’ physical literacy is positively associated with their children’s physical activity, directly and indirectly, through supportive behavior.

**Methods:**

In this cross-sectional study, we analyzed data from 2,000 parents (mean age, 40.2 ± 5.4 years) of children aged 4–9 years from the follow-up survey of the Japan Sports Agency Project for Promoting Physical Activity Habits from Early Childhood. Parents’ physical literacy was assessed using the Physical Literacy for Life self-assessment tool, parental support was assessed using five validated items, and children’s physical activity was assessed using two parent-reported questions. Structural equation modeling with bootstrapping and bias-corrected confidence intervals (5,000 samples) was used to examine the direct and indirect associations among these variables.

**Results:**

Structural equation modeling demonstrated an acceptable model fit (comparative fit index = 0.980, goodness-of-fit index = 0.981, adjusted goodness-of-fit index = 0.968, root mean square error of approximation = 0.047). Parents’ physical literacy was positively associated with both parental support (β = 0.28, *p* < 0.01) and children’s physical activity (β = 0.09, *p* < 0.01). Parental support was also associated with children’s physical activity (β = 0.60, *p* < 0.01).

**Conclusions:**

Parents’ physical literacy was positively associated with parental support and children’s physical activity, suggesting a mediating role of parental support in this relationship. Enhancing parents’ physical literacy may encourage supportive behaviors that contribute to children’s physical activity through family-based approaches.

## Background

 Physical activity has multifaceted health benefits, including the prevention of cardiovascular disease, weight management, and enhancement of mental health [1]. These also apply to children, with physical activity contributing not only to physical health but also to enhanced cognitive function and improved academic performance [2–6]. Physical activity and sedentary behavior patterns in early childhood (< 6 years of age) track into middle childhood (6–12 years) [7], and physical activity during adolescence is associated with physical activity levels in adulthood [8]. Improving children’s physical activity levels is important for their immediate and long-term health, making it an essential public health priority. Informed by current scientific evidence, the World Health Organization has established guidelines recommending at least 60 min of moderate-to-vigorous physical activity daily for children aged 5–17 years [9]. However, only 27–33% of children worldwide comply with these guidelines [10].

Since children spend much of their time with their families, family-based interventions are considered a critical approach to promoting physical activity [11, 12]. Of particular interest is parental support, which influences children’s physical activity levels through indirect aspects such as encouragement, involvement, and promotion [13–16], as well as direct actions such as transportation and co-participation in activities [17, 18]. Therefore, parental support is considered an important factor in promoting children’s physical activity.

A concept that has received increasing attention in relation to physical activity is physical literacy, which has been proposed as a potential factor in promoting health and preventing diseases [19, 20]. The International Physical Literacy Association defines it as “the motivation, confidence, physical competence, knowledge, and understanding to value and take responsibility for engagement in physical activities for life” [21], representing a comprehensive framework for promoting lifelong participation in physical activity. Physical literacy includes physical (abilities and skills), emotional (confidence and motivation), cognitive (knowledge and strategies), and social (ethics and social skills) domains [20, 22, 23]. Notably, individuals with high physical literacy may engage in physical activities throughout their lives [23]. Previous studies on physical literacy have demonstrated a correlation with health indicators, including physical activity, body composition, blood pressure, and health-related quality of life [24, 25].

Recent research by Ha et al. (2022) found that parents’ physical literacy is associated with their children’s physical activity values [26]. Additionally, quasi-experimental research on parents’ physical literacy has shown positive associations between parents’ physical literacy and parent–child participation in physical activity [27]. Nonetheless, the structure of the relationship between parents’ physical literacy and children’s physical activity (i.e., whether it is direct or mediated) has not been clarified. To better understand this relationship, we examined the association between parents’ physical literacy, parental support, and children’s physical activity. We hypothesized that parents’ physical literacy is positively associated with children’s physical activity, with supportive behaviors mediating this relationship.

## Methods

### Data source and study sample

We used data from a longitudinal survey conducted by the Japan Sports Agency as part of the Project for Promoting Physical Activity Habits from Early Childhood (https://www.mext.go.jp/sports/b_menu/sports/mcatetop03/list/1396909_00001.htm). The project was designed to collect comprehensive information on children’s physical activity and related family factors across a national sample in Japan, where national data on early childhood physical activity are limited. We conducted an initial survey from December 16 to 26, 2022, and follow-up surveys from December 1 to 4, 2023, and December 1 to 16, 2024. We analyzed the data collected from December 1 to 4, 2023, because the survey contained all the information necessary to achieve the study’s purpose.

The longitudinal survey was conducted by an internet panel company (http://www.myvoice.co.jp). A screening survey was sent to 235,546 individuals between December 8 and 23, 2022, of whom 42,554 met the inclusion criteria. Of these, 13,135 were invited to participate in the first-year survey, and 6,576 responded. After excluding 1,323 participants with missing or inconsistent data, the final sample for the first-year survey was determined (5253 participants). Participants were recruited based on the demographic characteristics of the Japanese population.

The second-year survey was conducted from December 1 to 4, 2023. The company sent email invitations to 4,048 Japanese parents who had completed the first-year survey (display rate: 77.06%), and 2,262 participants completed the survey (participation rate: 55.88%). The panel company excluded 262 respondents based on their screening criteria, including those who reported not having children or provided missing or inconsistent data.

Ultimately, 2,000 parents of children aged 4–9 years who consented to the study procedures and completed the questionnaires were included in the analysis. All questionnaires were completed by parents, and all information regarding the children was reported by the parents. This study was a secondary analysis of survey data collected as part of the Japan Sports Agency project, with permission from the agency. The use of these data for secondary analysis was approved by the Juntendo University School of Health and Sports Science and Graduate School of Health and Sports Science Research Ethics Committee (no. 2024-69).

### Measures

#### Parental support for children’s physical activity

Parental support for children’s physical activity was assessed by five questions established previously [30], and read as follows: “encouraged their child to do physical activities or play sports,” “done a physical activity or played sports with their child,” “provided transportation so their child could go to a place where he or she can do physical activities or play sports,” “watched their child participate in physical activity or sports,” and “told their child that physical activity is good for his or her health.” For these questions, parents reported weekly frequency on a 5-point scale (never, 1–3 times a month, 1–2 times a week, 3–6 times a week, and every day). Here, responses were scored as 0 points for “never,” 1 point for “1–3 times a month,” 2 points for “1–2 times a week,” 3 points for “3–6 times a week,” and 4 points for “every day.”

#### Children’s physical activity

Parent-reported child physical activity was assessed using two questions. The first question was “How much physically active play does your child engage in?” with the following four response options: Not at all, Only a little, Often, and Very often. The second question was “On how many days during the last 7 days did your child engage in physical activity (including exercise/sports, physical play, and walking to and from school) for a total of at least 60 min per day?” with a range of 0–7 days.

#### Demographic information

The parents’ and children’s ages and sex were examined. Annual household income was determined using the following categories: <2, 2–3, 3–4, 4–5, 5–6, 6–7, 7–8, 8–9, 9–10, and > 10 million yen.

### Data analysis

Descriptive analysis was performed to summarize the variables (n, %, mean, standard deviation, range, median, skewness, and kurtosis). Spearman correlation analysis was conducted to explore the initial bivariate relationships among the major variables. Structural equation modeling was used to examine the relationships between parents’ physical literacy, parental support for children’s physical activity, and children’s physical activity. The analysis modeled the major variables as latent and the corresponding items as observed. All the observed variables were treated as continuous indicators in the analysis. The measurement model incorporated three latent variables: parents’ physical literacy (four domains), parental support (five items), and children’s physical activity (two indicators). The multiple-indicator model was created and analyzed with the following paths: “Parents’ physical literacy → Parental support → Children’s physical activity” and “Parents’ physical literacy → Children’s physical activity.” Parameter estimates were calculated using the maximum likelihood. To examine the significance of mediating effects and address departures from multivariate normality, 95% bias-corrected confidence intervals (CIs) were estimated from 5,000 bootstrap resamples [31, 32]. The paths were considered significant if the 95% CI did not include zero [31]. Model fit was assessed using indices such as the comparative fit index (CFI), goodness-of-fit index (GFI), adjusted goodness-of-fit index (AGFI), and the root mean square error of approximation (RMSEA). The CFI, GFI, and AGFI values ranged from 0 (poor fit) to 1 (good fit). RMSEA value ≤ 0.08 is considered acceptable, while a value ≤ 0.05 indicates a very good fit [33]. For relative comparison among multiple models, the Akaike information criterion (AIC) was used [34], with lower values indicating better-fitting models. All coefficients reported in our analyses were thus standardized. All statistical analyses were performed using SPSS Statistics version 28 or SPSS AMOS version 26 (IBM Corp., Armonk, NY, USA). Significance was set at *p* < 0.05 for all analyses.

#### Parents’ physical literacy

The Physical Literacy for Life self-assessment tool (PL4L) was used to assess parents’ physical literacy [28]. A previous study examined the factor structure of the tool and found satisfactory internal consistency (α = 0.92) [29]. The tool comprised four domains: The physical domain included six items on the ability to perform physical activities (strength, stamina, and skills); the emotional domain consisted of four items, motivation and confidence to participate in regular physical activity and the regulation of physical and emotional states during physical activity; the cognitive domain included three items on knowledge, rules, and strategies for physical activity; and the social domain consisted of three items assessing interactions with others, such as ethics and collaboration in physical activity. Each item had three response options (levels 1–3), and parents were asked to select the level that best suited them. In this study, responses were scored as 1 point for level 1, 2 points for level 2, and 3 points for level 3. The resulting score ranges were 6–18 (physical domain), 4–12 (emotional domain), 3–9 (cognitive domain), 3–9 (social domain), and 16–48 (total score).

## Results

The study included 546 men (fathers; 27.3%) and 1,452 women (mothers; 72.6%), with a mean age of 40.2 ± 5.4 years. The participants comprised 1,023 boys (51.2%) and 977 girls (48.9%), with a mean age of 6.7 ± 1.6 years. The median annual household income was 7–8 million yen, and 53.7% of the participants had two children in their household (Table 1). The mean total scores for parents’ physical literacy and parental support for children’s physical activity were 34.4 ± 5.3 and 6.4 ± 4.0, respectively. For physically active play, parents reported that 72 (3.6%), 701 (35.1%), 889 (44.5%), and 338 (16.9%) children engaged in “Not at all,” “Only a little,” “Often,” and “Very often,” respectively. The average number of days that the children engaged in physical activity was 3.5 ± 2.3 (Table 2).

**Table 1:** Participants’ characteristics

		*n*		%
Parents’ sex				
Men		546		27.3
Women		1452		72.6
Prefer not to answer		2		0.1
Parents’ age	mean ± SD 40.2 ± 5.4			
25–29 years old		36		1.8
30–39 years old		905		45.3
40–49 years old		977		48.9
50–59 years old		78		3.9
60–62 years old		4		0.2
Children’ sex				
Boys		1023		51.2
Girls		977		48.9
Children’ age	mean ± SD 6.7 ± 1.6			
4 years old		217		10.9
5 years old		328		16.4
6 years old		305		15.3
7 years old		397		19.9
8 years old		411		20.5
9 years old		342		17.1
Annual household income				
< 2 million yen		80		4.0
2–3 million yen		67		3.4
3–4 million yen		152		7.6
4–5 million yen		198		9.9
5–6 million yen		270		13.5
6–7 million yen		226		11.3
7–8 million yen		215		10.8
8–9 million yen		171		8.6
9–10 million yen		137		6.9
> 10 million yen		265		13.3
Prefer not to answer		219		11.0

*SD* Standard deviation

**Table 2:** Descriptive statistics of the major variables

		Mean SD	Median	Skewness	Kurtosis
Parents’ physical literacy					
Physical domain (range: 6–18)		13.0 ± 1.9	12.0	1.085	1.791
Emotional domain (range: 4–12)		8.6 ± 1.8	9.0	0.039	0.117
Cognitive domain (range: 3–9)		6.4 ± 1.4	6.0	0.230	0.302
Social domain (range: 3–9)		6.4 ± 1.2	6.0	0.470	1.158
Total score (range: 16–48)		34.4 ± 5.3	33.0	0.652	1.132
Parental support for children’s physical activity					
Encourage		1.4 ± 1.1	1.0	0.378	−0.620
Play sports with their child		1.0 ± 0.9	1.0	0.729	0.314
Provided transportation		1.3 ± 0.9	1.0	0.353	−0.152
Watch child participate		1.4 ± 1.0	1.0	0.480	0.003
Tell good for health		1.3 ± 1.2	1.0	0.691	−0.354
Total score (range: 0–20)		6.4 ± 4.0	6.0	0.397	−0.074
Children’s physically active play					
Not at all	n (%)	72 (3.6)			
Only a little	n (%)	701 (35.1)			
Often	n (%)	889 (44.5)			
Very often	n (%)	338 (16.9)			
Children engaged in physical activity days		3.5 ± 2.3	4.0	−0.061	−1.213
0	n (%)	250 (12.5)			
1	n (%)	249 (12.5)			
2	n (%)	247 (12.4)			
3	n (%)	235 (11.8)			
4	n (%)	163 (8.2)			
5	n (%)	472 (23.6)			
6	n (%)	146 (7.3)			
7	n (%)	238 (11.9)			

*SD* Standard deviation

Table 3 presents the results of the Spearman correlation analysis performed to examine the initial bivariate relationships among the major variables. The correlation coefficients ranged from 0.11 to 0.40 and were all significant. The highest correlation coefficient (0.40) was observed between parental support and physically active play.

**Table 3:** Spearman Correlation Matrix for the Major Variables and Demographic Information

	#1		#2		#3		#4		#5	#6	#7	#8	#9
#1 Parent’s sex	1												
#2 Parent’s age	– 0.27**		1										
#3 Children’s sex	0.05*		0.01		1								
#4 Children’s age	0.01		0.01		0.00		1						
#5 Annual household income	– 0.09**		0.17**		– 0.02		0.02		1				
#6 Parents’ physical literacy	– 0.24**		0.08**		0.04		– 0.03		0.07**	1			
#7 Parental support for children’s physical activity	– 0.04		0.00		– 0.03		0.05**		0.13**	0.27**	1		
#8 Children’s physically active play	0.07**		– 0.10**		– 0.04		– 0.04		0.07**	0.19**	0.40**	1	
#9 Children engaged in physical activity days	0.11**		– 0.03		– 0.04		– 0.05*		0.06*	0.11**	0.28**	0.40**	1

*p < 0.05, **p < 0.01

**Fig. 1:**
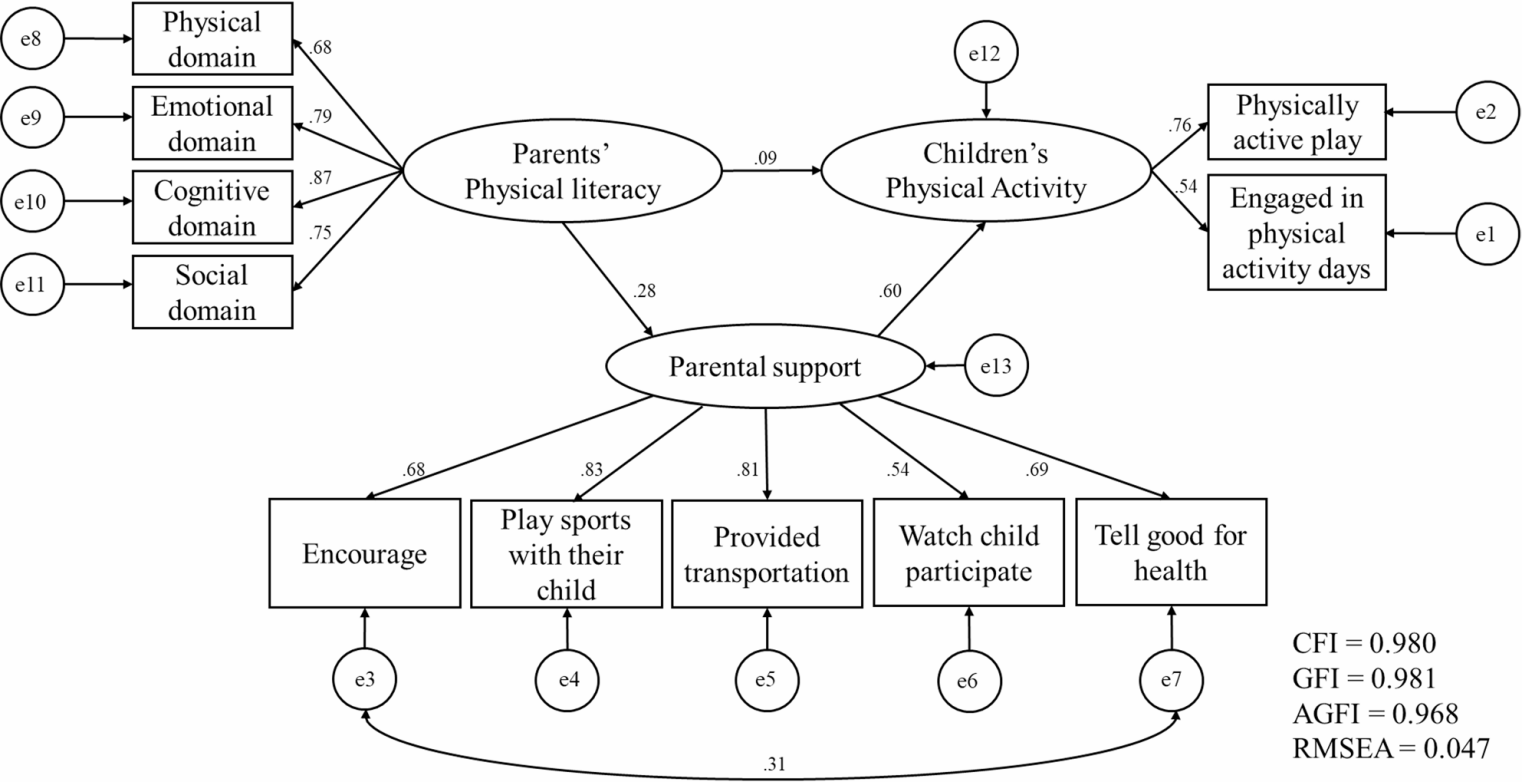
The relationships between parents’ physical literacy, parental support, and children’s physical activity. Goodness-of-fit indices (GFI, AGFI, CFI) suggest a better model fit with higher values, whereas RMSEA indicates a better fit with lower values. The standardized path coefficients are shown. All paths were significant (p < 0.01). Bootstrap estimates (95% bias-corrected CIs) indicated that parents’ physical literacy was positively associated with children’s physical activity (β = 0.09, 95% CI [0.03, 0.15]), as well as with parental support (β = 0.28, 95% CI [0.23, 0.34]). Parental support was positively associated with children’s physical activity (β = 0.60, 95% CI [0.54, 0.66]). CI, confidence interval; CFI, comparative fit index; GFI, goodness-of-fit index; AGFI, adjusted goodness-of-fit index; RMSEA, root mean square error of approximation

The fit between the initial model and the data was acceptable, with CFI = 0.961, GFI = 0.966, AGFI = 0.946, and RMSEA = 0.065. Nonetheless, the parental support items “encourage” and “tell good for health” involve verbally encouraging or advising the child, suggesting similarity in the type of behavior assessed. Therefore, it is reasonable to assume an error covariance between these two items. After accounting for error covariance, the modified model showed an improved fit, with CFI = 0.980, GFI = 0.981, AGFI = 0.968, and RMSEA = 0.047 (Fig. 1). The AIC used for relative comparisons among the multiple models improved from 432.549 in the initial model to 266.726 in the modified model. In the latter, all paths were significant. The coefficients for parents’ physical literacy to children’s physical activity, parents’ physical literacy to parental support, and parents’ support to children’s physical activity were 0.09 (95% CI [0.03, 0.15]), 0.28 (95% CI [0.23, 0.34]), and 0.60 (95% CI [0.54, 0.66]), respectively. Based on these results, the direct effect of parents’ physical literacy on children’s physical activity was 0.09. In addition, an indirect effect of 0.17 (0.28 × 0.60) was observed through parental support, resulting in a total effect of 0.26.

Overall, the results showed that parents’ physical literacy was positively associated with children’s physical activity, with parental support mediating this relationship, consistent with the hypothesized model.

## Discussion

Physical literacy has gained attention as a health-related concept [19], as reflected in the increasing number of published studies [35]. Conversely, due to its conceptual closeness to physical education [36, 37], previous studies have focused on children and adolescents [38]. Therefore, little attention has been paid to family-based physical literacy, in which parents play a key role in children’s lives. Our study findings contribute to physical literacy research by focusing on the home context, particularly the association between parents’ physical literacy and children’s physical activities. Additionally, the results suggest that parent- and family-focused interventions in physical literacy may promote children’s physical activity, underscoring the importance of advancing physical literacy in school and family contexts. Expanding research on home-based physical literacy alongside school-based studies has the potential to provide a more comprehensive understanding of the strategies to promote children’s physical activity.

In this study, parents’ physical literacy was positively associated with parental support, including behaviors such as verbal encouragement, co-participation in physical activity, and logistical and informational support (β = 0.28). Similarly, another study showed that parents with higher physical literacy were more likely to value their own physical activity and engage in autonomy-supportive practices for their children’s physical activity [26]. Research focusing on parents’ physical literacy has demonstrated that it is associated with increased co-physical activity with their children [27], and parents who participated in physical literacy workshops shared what they learned with their children at home [39], both of which may reflect parental support. Thus, taken together with the present findings, parents’ physical literacy may promote children’s physical activity, thereby supporting their engagement.

Among the associations examined, the strongest association observed was between parental support and children’s physical activity (β = 0.60), corroborating previous research suggesting that parental support acts as a potential facilitator of physical activity [16, 17]. Such support provided by parents who are involved in their children’s daily lives may influence children’s physical activity by shaping opportunities and engagement in daily activities. Therefore, interventions targeting parental support may help promote physical activity among children.

The direct path from parents’ physical literacy to children’s physical activity was significant (β = 0.09); however, the effect size was small. This suggests that parents’ physical literacy is just one of the many determinants of children’s physical activity, and its direct influence should be interpreted with caution. Previous research has suggested that short-term interventions aimed at improving parents’ physical literacy may not be sufficient to influence children’s activity levels directly [27]. Given the potential delay in observable effects in children, longitudinal and long-term studies are needed to better understand these relationships. Taken together with the present findings, interventions aimed at improving physical literacy and facilitating daily parental support for children’s physical activity are warranted.

This study is not without limitations. First, because it was a cross-sectional study, establishing causal relationships among parents’ physical literacy, parental support, and children’s physical activities was not possible. Future studies should address this issue using longitudinal designs and intervention studies targeting parents’ physical literacy to better understand how parents’ physical literacy relates to parental support and children’s physical activity. Furthermore, the participants in this study were limited to those registered with an internet panel survey company [40]. The analysis included only those who participated in the second-year survey among the sample drawn to reflect Japanese demographics, which may affect the extent to which the results can be generalized. Finally, because the data were based on parent-reported questionnaires, they may have been affected by social desirability and recall biases [41]. Future research could benefit from using objective measures, such as wearable devices, to provide more accurate estimates of children’s physical activity.

## Conclusions

Parents’ physical literacy was positively associated with parental support and children’s physical activity, suggesting a mediating role of parental support in this relationship. These findings suggest the importance of parents’ physical literacy as a pathway to children’s physical activity through daily supportive behaviors at home. Future research should focus on longitudinal and intervention studies to clarify causal relationships and identify effective family-based strategies for promoting children’s physical activity, including the role of parents’ physical literacy and parental support.

## Data Availability

The datasets used and/or analyzed in this study are available from the corresponding author upon reasonable request.

## Abbreviations

CFI: Comparative fit index
GFI: Goodness of fit index
AGFI: Adjusted goodness of fit index
RMSEA: Root mean square error of approximation
AIC: Akaike information criterion
